# Study on the Pyrolysis and Fire Extinguishing Performance of High-Temperature-Resistant Ultrafine Dry Powder Fire Extinguishing Agents for Aviation Applications

**DOI:** 10.3390/molecules29153500

**Published:** 2024-07-26

**Authors:** Zhixuan Wang, Yi Zhang, Yurong Liu, Jun Wang, Xia Zhou, Renming Pan

**Affiliations:** 1 School of Safety Science and Engineering (School of Emergency Management), Nanjing University of Science and Technology, Nanjing 210094, China; zx77.wang@foxmail.com (Z.W.); zhangyi0911@njust.edu.cn (Y.Z.); 320103010109@njust.edu.cn (Y.L.); lyn122417@163.com (J.W.); 2 School of Fashion and Textiles, The Hong Kong Polytechnic University, Kowloon, Hong Kong SAR 999077, China

**Keywords:** K-dawsonite dry powder, halon alternatives for aviation applications, hydrophobic and oleophobic characteristics, pyrolysis kinetics, fire extinguishing efficiency

## Abstract

Ultrafine KAl(OH)_2_CO_3_ dry powder (UDWP), as a novel high-temperature-resistant ultrafine dry powder fire extinguishing agent, has garnered significant attention in the field of aviation fire protection. However, its development has been hindered by its hydrophilicity, which leads to hygroscopicity, and its tendency for re-ignition due to oil deposition. Therefore, this study employs perfluorodecyltrimethoxysilane (PFDTMS) to modify the surface of UDWP, resulting in hydrophobic and oleophobic M-UDWP. The thermal stability and hydrophobicity of M-UDWP ensure its long-term stable storage in aircraft equipment compartments, thereby reducing aircraft maintenance costs. Additionally, its oleophobicity provides excellent anti-re-ignition performance, protecting aircraft power compartments from secondary fire damage. Energy-dispersive X-ray spectroscopy (EDS), Fourier-transform infrared spectroscopy (FTIR), and X-ray photoelectron spectroscopy (XPS) analyses indicate that the PFDTMS modifier was successfully grafted onto KAl(OH)_2_CO_3_. Furthermore, M-UDWP exhibits a three-stage thermal decomposition process. The first-stage decomposition can be regarded as a single-step reaction, and the calculated kinetic parameters provide accurate predictions. Thermogravimetric analysis-Fourier transform infrared spectroscopy-mass spectrometry (TG-FTIR-MS) results reveal that M-UDWP significantly produces H_2_O and CO_2_ during thermal decomposition, which is one of its core fire extinguishing mechanisms. For the combustion of #RP-3 and #RP-5 aviation kerosene, commonly found in aircraft engine nacelles, the extinguishing times required by M-UDWP are 243 ms and 224 ms, respectively, with minimum extinguishing concentrations (MEC) of 25.9 g/m^3^ and 23.4 g/m^3^, respectively. The study of M-UDWP’s thermal stability aids in understanding its storage stability under high-temperature conditions and its fire extinguishing mechanisms in fire zones. Moreover, the research findings suggest that M-UDWP has the potential to replace Halon 1301 in aircraft engine nacelles.

## 1. Introduction

Following the 1987 Montreal Protocol, it was discovered that halogenated fire suppressants severely damage the atmospheric ozone layer [1]. Researchers have begun searching for alternatives to halogen-free flame retardants [2,3], including those that can be used in fire extinguishers [4,5]. The European Aviation Safety Agency (EASA) mandated in its 2015 CS-25 amendment the gradual phase-out of halon fire suppressants in engine compartments by 2041, necessitating their replacement [6]. Foam, water mist, aerosol, and cold aerosol extinguishing agents are all viable alternatives to Halon extinguishing agents. However, water-based extinguishing agents like foam and water mist and their corresponding extinguishing devices have excessive weight and volume, and these water-based agents bring about a series of corrosion issues [7]. These disadvantages significantly increase the cost of civil aviation transportation, making them unsuitable as Halon replacements on aircraft. Aerosol fire extinguishing agents have ultra-high fire extinguishing efficiency, but their release process is accompanied by high temperatures, inadvertently introducing new risks to aircraft operations [8]. Therefore, they are not chosen as Halon replacement extinguishing agents on aircraft. Cold aerosol fire extinguishants, also known as ultrafine dry powder extinguishants, consist of particles smaller than 20 μm. These extinguishants possess zero ozone depletion potential (ODP) and zero global warming potential (GWP), thus neither depleting the ozone layer nor contributing to global warming [9,10]. Due to their excellent fire suppression capabilities, they are recommended by Kydex Defense as ideal substitutes for halon fire suppressants in aircraft engine nacelles [11]. Commercial ultrafine dry powders containing NaHCO_3_ and NH_4_H_2_PO_4_ decompose at 90 °C and 150 °C, respectively [4,12]. However, the JTCG report states that aero-engine nacelle extinguishing agents require a chemical/physical decomposition weight loss of no more than 5% wt over the temperature range of −55 °C to 260 °C [13,14]. The decomposition temperatures of BC and ABC ultrafine dry powder extinguishants, primarily composed of NaHCO_3_ and NH_4_H_2_PO_4_, are too low for long-term storage in aircraft equipment compartments.

K-dawsonite (KAl(OH)_2_CO_3_) has long been utilized in various fields due to its outstanding thermal insulation, low alkalinity, and unique chemical properties. It serves as an antacid, corrosion inhibitor, solid-base catalyst, and precursor for novel ceramic materials. Additionally, the excellent thermal stability of KAl(OH)_2_CO_3_ makes it a viable flame-retardant filler [15,16,17,18]. Consequently, some researchers have developed ultrafine dawsonite powder (UDWP) fire extinguishants using KAl(OH)_2_CO_3_ as the active component [19]. Studies have shown that UDWP extinguishes fires by decomposing to generate water vapor, carbon dioxide, and absorbing heat while capturing active free radicals produced during combustion [20]. This effectively suppresses flames, with fire suppression efficiency surpassing that of traditional ultrafine dry powders. UDWP decomposes around 278 °C, producing carbon dioxide and water as the main decomposition products. Its good thermal stability and excellent fire suppression performance indicate potential as a fire suppressant for aircraft engine nacelles.

However, UDWP lacks oleophobic properties, making it difficult to meet the re-ignition resistance standards required for aviation fire suppressants. To meet aviation standards, researchers such as Ning [21], Guo [22], Zhou [23], and Zhang [24] have modified BC dry powder to be hydrophobic and oleophobic. They employed low surface energy modifiers to enhance the flowability of ultrafine BC dry powder, enabling it to float on the surface of hydrocarbon fuels like aviation kerosene and form a flame-retardant layer. This effectively reduces the volatilization of combustible vapors and prevents fuel re-ignition. Zhao [25] surface-modified ultrafine ammonium polyphosphate dry powder extinguishants, which also exhibited fire suppression efficiency and re-ignition resistance far exceeding those of commercial ultrafine dry powder extinguishants.

In this study, perfluorodecyltrimethoxysilane (PFDTMS) was used to modify UDWP, producing hydrophobic and oleophobic ultrafine dawsonite powder extinguishants (M-UDWP). The water and oil contact angles of M-UDWP were tested. SEM, XPS, and FTIR techniques were employed to verify the successful grafting of PFDTMS onto M-UDWP particles. Thermogravimetric analysis was used to study the thermal decomposition behavior of M-UDWP, and three model-free methods were utilized to calculate the kinetic parameters (activation energy, pre-exponential factor, and reaction model) of M-UDWP at different conversion rates. Real-time thermogravimetric analysis (TG), Fourier-transform infrared spectroscopy (FTIR), and mass spectrometry (MS) were used to analyze the volatile products during the thermal decomposition of M-UDWP, assessing the impact of the modified coating on the fire extinguishing efficiency. Finally, the fire suppression efficiency of M-UDWP against #RP-3 and #RP-5 aviation kerosene pool fires was tested in a 0.1 m^3^ confined space with obstacles. The re-ignition delay time (RTD) of M-UDWP was tested, and its fire suppression mechanism was analyzed.

## 2. Experimental Details

### 2.1. Materials

The primary active component of M-UDWP, KAl(OH)_2_CO_3_, was synthesized by our research team. The flow-aiding additives, including nano calcium carbonate, muscovite, and talc powder, were procured from Shanghai Macklin Biochemical Co., Ltd. (Shanghai, China). The solvents used in the preparation, including deionized water (H_2_O), anhydrous ethanol (C_2_H_5_OH), and ammonia solution (NH_3_·H_2_O), were sourced from Nanjing Chemical Reagent Co., Ltd. (Nanjing, China). Perfluorodecyltrimethoxysilane (PFDTMS) was obtained from Bide Pharmatech Ltd., and tetraethyl orthosilicate (TEOS) was acquired from Sinopharm Chemical Reagent Co., Ltd. (Shanghai, China). The fuels used in the fire suppression experiments, including #RP-3 and #RP-5 aviation kerosene, were provided by the pharmacy of Nanjing University of Science and Technology. All materials and chemicals were used as received, without further purification.

### 2.2. Preparation of M-UDWP

**Scheme 1:**
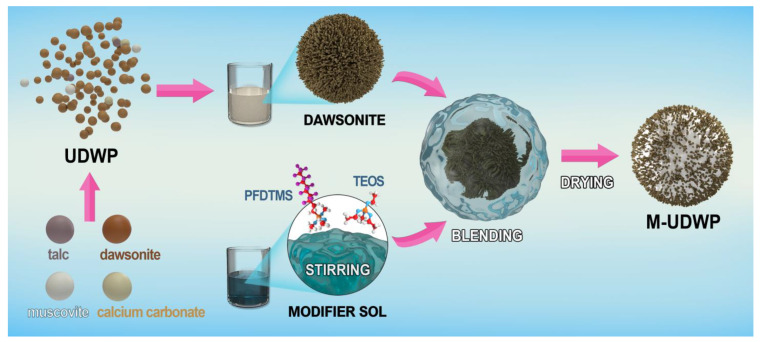
Preparation of UDWP and its modification into M-UDWP.

The preparation and modification process of M-UDWP is illustrated in Scheme 1. The M-UDWP samples were obtained through a two-step process. Ning [26] studied the dosage and usage methods of five additives—muscovite, talc, synthetic zeolite, activated clay, and nano calcium carbonate—concerning their effects on the flowability of BC dry powder. We specifically optimized and simplified the formulation for UDWP modification needs, selecting three effective additives—muscovite, nano calcium carbonate, and talc—to ensure a high proportion of core active components in UDWP, thereby improving the fire extinguishing effectiveness of UDWP. In the first step, KAl(OH)_2_CO_3_, muscovite, nano calcium carbonate, and talc were thoroughly mixed in a ratio of 90:3:4:3 to prepare the UDWP. In the second step, following Guo’s method for modifying ultrafine BC dry powder [22], the UDWP was modified using PFDTMS to obtain M-UDWP. First, 150 g of UDWP were dispersed in a solution containing 150 mL of deionized water and 150 mL of anhydrous ethanol, and mechanically stirred at room temperature for 60 min. Subsequently, 10 g of TEOS, 15 g of PFDTMS, and 20 mL of ethanol were mixed, and NH_3_·H_2_O was added to adjust the pH to 9–10. The mixture was thoroughly stirred to initiate hydrolysis and condensation reactions, forming a stable, transparent sol in the solution. The transparent sol was then added to the UDWP suspension and mechanically stirred at 45 °C for 12 h. The resultant product was dried in a forced-air drying oven at 60 °C for 10 h to finally obtain M-UDWP.

### 2.3. Thermal Analysis Experiment

Thermogravimetric analysis (TGA) and differential scanning calorimetry (DSC) were performed using a METTLER TOLEDO TGA/DSC3+ analyzer (Shanghai, China). This study focused on the thermal degradation behavior of M-UDWP samples [27], with each sample weighing approximately 1.8 mg. The analysis was performed under a nitrogen flow (99.99% purity) at a flow rate of 100 mL/min and heating rates of 15, 20, 25, and 30 K/min. The temperature range corresponding to the heating rates was selected from 320 K to 1050 K. An online TG-FTIR-MS instrument (STA449F3-Nicolet iS50-QMS403D, Thermo Fisher Scientific, Waltham, MA, USA) was employed to detect volatile compounds generated during thermal degradation in a nitrogen atmosphere (250 mL/min). The heating rate of 10 K/min and the temperature range used in the online TG-FTIR-MS analysis were consistent with those used in the thermogravimetric analysis.

Estimating kinetic parameters can provide valuable insights into the thermal degradation of materials and can be utilized to predict material behavior under general thermal conditions. The activation energy and conversion mechanisms of M-UDWP were determined using non-isothermal kinetic methods. The thermal decomposition rate of condensed phase materials can typically be mathematically described by Equations (1) and (2):

(1) $$\frac{d\alpha }{dT}=\frac{A}{\beta }\exp(-\frac{E_{\alpha }}{RT})f(\alpha )$$

(2) $$g(\alpha )=\int_{0}^{\alpha }\frac{d\alpha }{f(\alpha )}=\frac{A}{\beta }\exp\left(-\frac{E_{\alpha }}{RT}\right)dT=\frac{AE_{\alpha }}{\beta R}\int_{y_{\alpha }}^{\infty }\frac{\exp\left(-y\right)}{y^{2}}dy=\frac{AE}{\beta R}p(y)$$

In this context, *α*, *A*, *β*, *E_α_*, and R represent the conversion rate, pre-exponential factor (min^−1^), heating rate (K/min), activation energy (J/mol), and universal gas constant (J/(mol·K)), respectively. The conversion rate *α* is defined as *α* = (*m*_0_ − *m*)/(*m*_0_ − *m*_∞_), where *m*_0_ denotes the initial mass and m_∞_ denotes the final mass. The reaction models *f*(*α*) and *g*(*α*) are expressed in differential and integral forms, respectively. Table S1 lists 36 common solid-state thermal decomposition reaction models and their mechanisms.

Three model-free methods, namely the Distributed Activation Energy Model (DAEM), the Flynn-Wall-Ozawa (FWO) method, the Starink method, as well as a model-fitting method and the Coats-Redfern (CR) method, were employed for kinetic analysis. DAEM [28,29] is considered an effective and accurate method for simulating complex combustion reactions involving multiple parallel first-order irreversible reactions. The FWO method [30,31] allows for the accurate assessment of activation energy and reaction mechanisms for solid-state combustion by avoiding the errors and limitations inherent in conventional analysis methods. The Starink method [32], derived from the FWO method, is also regarded as effective and precise, with the activation energy (*E_α_*) calculated based on the slope of the best linear regression model of ln(*β*/*T*^1.92^) versus 1/*T*. The CR method [28] is a popular model-fitting method used to estimate kinetic mechanisms and parameters (*E* and *A*). In this study, the thermal decomposition process was analyzed using the equations of DAEM, FWO, and CR, as shown in Equations (3)–(6):

DEAM:

(3) $$\ln\left(\frac{\beta }{T_{\alpha }^{2}}\right)=\ln\left(\frac{A_{\alpha }R}{E_{\alpha }g(\alpha )}\right)+0.6575-\frac{E_{\alpha }}{RT_{\alpha }}$$

FWO:

(4) $$\ln(\beta )=\ln\left(\frac{A_{\alpha }R}{E_{\alpha }g(\alpha )}\right)-5.331-1.052(\frac{E_{\alpha }}{RT_{\alpha }})$$

Starink:

(5) $$\ln\left(\frac{\beta }{T_{\alpha }^{1.92}}\right)=\ln\left(\frac{A_{\alpha }R}{E_{\alpha }g(\alpha )}\right)-0.312-1.0008(\frac{E_{\alpha }}{RT_{\alpha }})$$

CR:

(6) $$\ln\left(\frac{g(\alpha )}{T_{\alpha }^{2}}\right)=\ln\left(\frac{A_{\alpha }R}{\beta E_{\alpha }}\right)-\frac{E_{\alpha }}{RT_{\alpha }}$$

The feasibility and direction of the heat conversion process, along with the energy changes occurring during it, can be evaluated and measured through key thermodynamic parameters: enthalpy (Δ*H*, J/mol), Gibbs free energy (Δ*G*, J/mol), and entropy (Δ*S*, J/(mol·K)). The specific formulas for calculating Δ*H*, Δ*G*, and Δ*S* are presented in Equations (7)–(9), where *K_B_* is the Boltzmann constant and *h* is the Planck constant:

(7) $$\Delta H=E_{\alpha }-RT_{m}$$

(8) $$\Delta G=E_{\alpha }+RT_{m}\ln(\frac{K_{B}T_{m}}{hA_{\alpha }})$$

(9) $$\Delta S=\frac{\Delta H-\Delta G}{T_{m}}$$

### 2.4. Fire Extinguishing Tests

**Figure 1:**
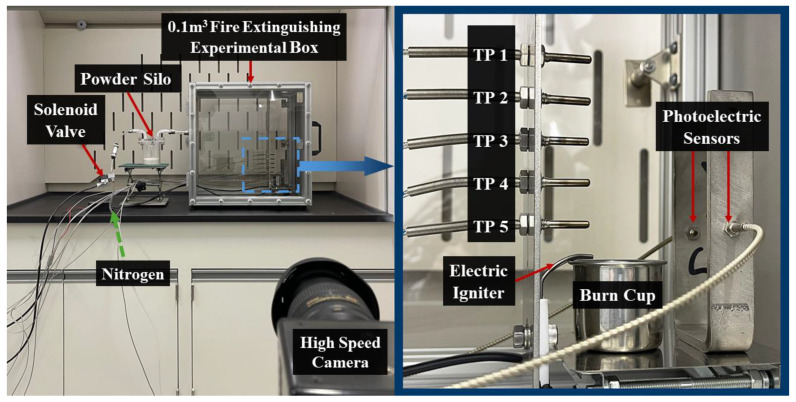
Fire extinguishment experimental setup.

In this study, a full-scale fire suppression experiment was conducted within a confined space of 0.1 m^3^. The experimental setup is illustrated in Figure 1. The front of the experimental chamber is fitted with quartz glass for real-time observation and recording of the test process, ensuring a clean testing environment and the safety of personnel. Five thermocouples, designated as TP 1 through TP 5, were positioned along the vertical axis directly above the circular burn cup, spaced 20 mm apart, with TP 1 located at the cup rim. An 18 kV arc igniter was placed 2 mm above the rim of the burn cup. Photodetectors (FU-84C, Keyence, Osaka, Japan) were positioned 20 mm on either side of the burn cup to measure the optical density of the powdered aerosol in real-time and calculate the minimum extinguishing concentration (MEC) of the ultrafine dry powder extinguishing agent.

To ensure precise measurement of the critical extinguishing concentration, a 6 cm wide baffle was installed between the nozzle and the flame. In an aircraft engine compartment, thermal insulation barriers prevent the direct path of the extinguishing agent to the fire source. The extinguishing agent must circumvent these physical obstructions to reach the fire source. Incorporating a baffle within the experimental chamber mimicked these obstacles, preventing the extinguishing agent from directly impacting the flame and thus approximating real fire scenarios more closely.

When setting up the fuel, 30 mL of deionized water was placed at the bottom of the burn pan before adding the fuel to prevent the fuel from boiling. In the fire suppression experiments with #RP-3 and #RP-5 aviation kerosene, 15 mL of aviation kerosene was added to the water’s surface, followed by 5 mL of n-heptane to facilitate ignition. This mixture was pre-ignition for 180 s, during which the chamber door was opened to ensure sufficient fuel combustion. The chamber door was closed 5 s before the extinguishing agent was discharged to ensure the experiment was conducted within a confined space. The fire suppression process was recorded using a high-speed camera (FASTCAM Mini UX, Photron, Tokyo, Japan). In configuring the ultrafine dry powder release mechanism, a specified mass of ultrafine dry powder extinguishing agent was placed into a sealed canister with a volume of 377 mL. This canister was connected to a nitrogen gas source via a solenoid valve and a precision pressure regulator, with the nitrogen pressure meticulously controlled at 0.50 MPa.

### 2.5. Characterizations

To evaluate the oleophobic and hydrophobic properties of the samples, contact angle measurements were conducted using an XG-CAME (SUNZERN, Shanghai, China) powder contact angle meter. Comprehensive characterization of the samples’ crystalline structure and elemental morphology was performed using XPS (PHI QUANTERA II, ULVAC-PHI, Chigasaki, Japan). For accuracy, all spectra were calibrated to the C 1s peak at a binding energy of 284.5 eV. Additionally, FTIR analysis (Nicolet 8700, Thermo Fisher Scientific, Waltham, MA, USA) was employed to detect the functional group characteristics of the samples. During this process, the samples were placed on potassium bromide (KBr) pellets and scanned at a slow rate of 0.158 cm/s within the wavenumber range of 450–4000 cm^−1^, achieving a resolution better than 0.09 cm^−1^.

The microstructure of the sample surfaces was observed using SEM (JSM-7800F PRIME, JEOL, Tokyo, Japan). To obtain clear images, the sample powders were first dispersed in ethanol and subjected to ultrasonic agitation for 10 min. Finally, a thin layer of gold was sputter-coated onto the powders for 60 s to enhance image clarity and conductivity.

## 3. Results and Discussion

### 3.1. Characterization of the M-UDWP Sample

**Figure 2:**
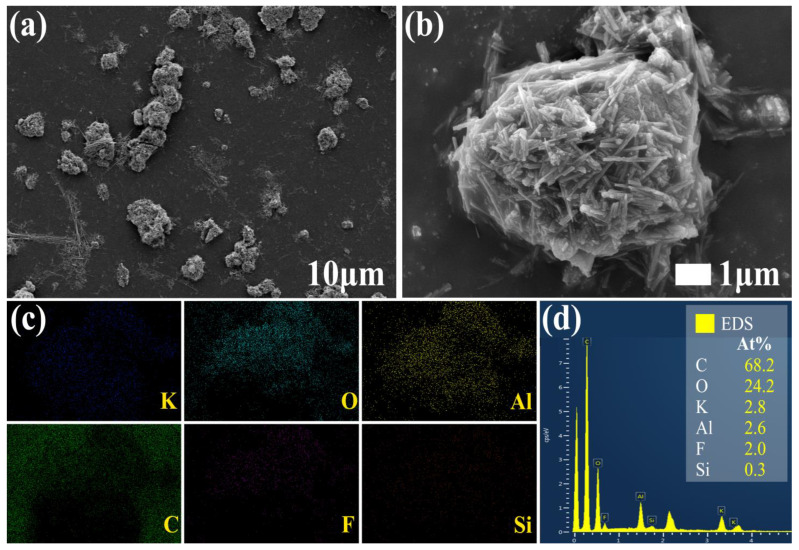
Surface morphology of M-UDWP (**a**,**b**) and EDS analysis of M-UDWP (**c**,**d**).

The scanning electron microscope images of M-UDWP are shown in Figure 2a,b. At a scale of 10 μm, significant agglomeration and adhesion among the powder particles were observed. The fibrous nature of KAl(OH)_2_CO_3_ crystals, characterized by a high aspect ratio and slender shape, predisposes them to aggregate through physical entanglement or electrostatic interactions. Following the introduction of the modifier, it became challenging for the fibrous powder surfaces to be completely exposed to the modifier due to their prior aggregated state. Consequently, the modifier tends to accumulate within the interstitial voids between the fibers rather than forming a uniformly thin film. At a scale of 1 μm, the modifier was observed to aggregate within the voids between fibers, causing the fiber powders to adhere into clusters and form spheroidal structures. However, a substantial number of fibrous structures or branches remained exposed outside the coating layer.

Figure 2c presents the EDS elemental mapping results for M-UDWP, revealing the presence of seven elements—C, O, K, Al, F, Au, and Si—on its surface, within the scanning area of Figure 2b. The Au on the powder surface originated from the gold sputtering applied to the sample. The presence of F and Si on the surfaces of both samples indicates the successful coupling of fluorosilane with TEOS, forming a modified coating layer. Figure 2d displays the overall spectrum of M-UDWP, with F content at 2.0% and Si content at 0.3%.

**Figure 3:**
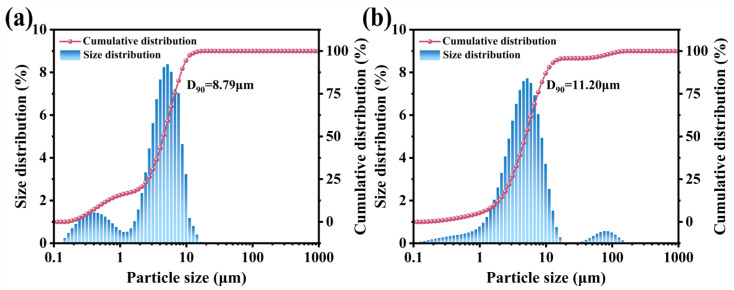
Particle size distribution and cumulative distribution of UDWP (**a**) and M-UDWP (**b**).

Conversely, as depicted in Figure 3a,b, the D_90_ particle size of UDWP was 8.29 μm, while that of the modified powder M-UDWP was 11.20 μm. The appearance of approximately 80 μm particles in M-UDWP is attributed to the addition of the modifier, which led to the adhesion and agglomeration of fibrous powder, forming larger particle clusters. The D_90_ of M-UDWP being significantly less than 15 μm confirms that M-UDWP still qualifies as an ultrafine dry powder extinguishing agent.

**Figure 4:**
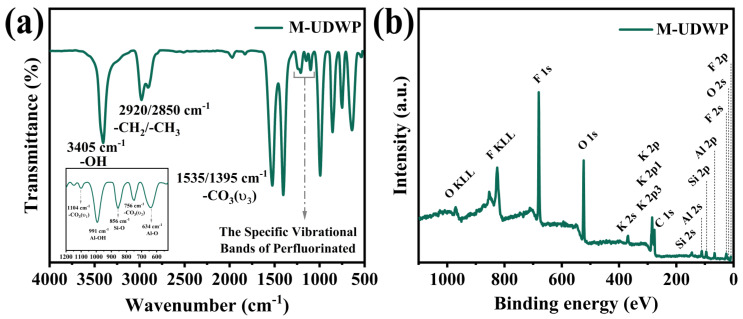
FTIR spectroscopy results of M-UDWP (**a**) and XPS full spectrum of M-UDWP (**b**).

The FTIR spectrum of M-UDWP, as illustrated in Figure 4a, displays all the characteristic absorption peaks of KAl(OH)_2_CO_3_. Specifically, the stretching vibration absorption peak of OH^−^ is located at 3405 cm^−1^, while the stretching vibration peaks of CO_3_^2−^ are distributed at 1395 cm^−1^, 1535 cm^−1^, and 1104 cm^−1^. The deformation vibration absorption peaks of CO_3_^2−^ appear at 866 cm^−1^ and 749 cm^−1^, the absorption peak of the Al-OH bond is observed at 991 cm^−1^, and the absorption peak of the Al-O bond is located at 652 cm^−1^. These peaks are consistent with the characteristic peaks of KAl(OH)_2_CO_3_ reported in the literature [19]. Furthermore, the characteristic peaks at 2920 cm^−1^ and 2850 cm^−1^ in M-UDWP are attributed to the symmetric and asymmetric -CH_2_ stretching vibrations of PFTDMS alkyl chains [33]. Similarly, the perfluorinated chain characteristic vibration bands are observed in the 1245–1018 cm^−1^ range, with distinct peaks at 1205 cm^−1^ and 1150 cm^−1^ corresponding to the -CF_2_^−^ and -CF_3_ functional groups, respectively [34]. The peak at 856 cm^−1^ is attributed to the Si-O stretching vibration. These findings indicate the successful grafting of PFTDMS onto M-UDWP [35,36].

Additionally, the XPS survey spectrum of the M-UDWP sample shown in Figure 4b distinctly identifies the absorption peaks of F 1s, F 2s, F 2p, Si 2s, and Si 2p. The aforementioned results further confirm that TEOS and PFDTMS, through hydrolysis and cross-linking reactions, have effectively coated the surface of M-UDWP particles, thereby imparting oleophobic and hydrophobic properties to the material.

**Figure 5:**
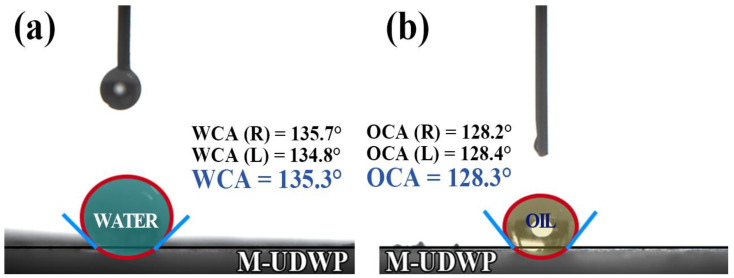
WCA (**a**) and OCA (**b**) test results of M-UDWP.

The contact angle is the angle formed by a liquid droplet on a solid surface, serving as an indicator of the surface’s hydrophobicity and oleophobicity [23]. Through modification with PFDTMS, the water contact angle (WCA) of the M-UDWP sample is measured at 135.3°, while the oil contact angle (OCA) is 128.3°, as shown in Figure 5a,b. Hydrophobicity and oleophobicity rates are defined as the ratio of the mass of powder floating on top of water/oil liquids to the original mass of the powder after stirring at 2000 rpm for 10 min. The original mass of the powder is denoted as M_0_, and the mass of the dried powder after stirring and drying is denoted as M_1_. The mass ratio data of M-UDWP before and after stirring in water and aviation kerosene are presented in Table 1, demonstrating that M-UDWP’s hydrophobicity rate exceeds 90% and the oleophobicity rate exceeds 85%. This is attributed to the high bond energy, low polarization, and high electronegativity of the C-F bonds in the long carbon-fluorine chains of PFDTMS [37]. The modifier self-assembles and coats the surface of M-UDWP powder particles; the fluorinated groups in the modifier tend to extend, accumulate, and orient towards the interface between the extinguishing agent and air, providing a “shielding effect” [38]. Additionally, the fluorocarbon chains exhibit low surface tension, which imparts excellent hydrophobic and oleophobic properties to the powder particles [39].

M-UDWP exhibits outstanding oleophobicity, ensuring that the powder can form a flame-retardant layer on the surface of unburned aviation kerosene after extinguishing a fire, thus preventing re-ignition. Furthermore, M-UDWP demonstrates good hydrophobicity, preventing moisture absorption and aggregation during long-term storage. These findings suggest that M-UDWP has potential applications in the fire protection of aircraft engine nacelles.

Thus, it is evident that the M-UDWP powder, modified by PFDTMS, exhibits both hydrophobic and oleophobic properties. Oleophobicity ensures that it can float on the fuel layer after extinguishing the flame, preventing re-ignition, while hydrophobicity ensures that the powder does not agglomerate due to water absorption and moisture uptake.

### 3.2. Kinetic Analysis

**Figure 6:**
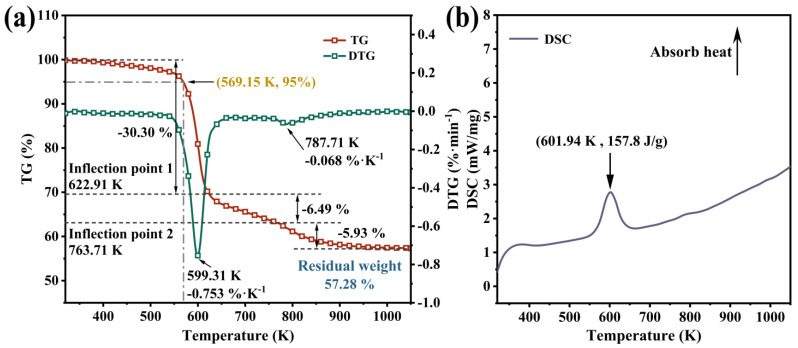
TGA and DTG test results for UDWP (**a**) and DSC test results for UDWP (**b**).

The TGA curve of M-UDWP is presented in Figure 6a. Under a nitrogen atmosphere, the temperature at which M-UDWP exhibits a 5% weight loss is 569.15 K, equivalent to 296 °C, meeting the requirements of the JTCG report. The weight loss percentage of M-UDWP at temperatures up to 373.15 K is approximately 0.14%, attributed to the evaporation of crystallization water molecules within M-UDWP. The DTG curve showcases two maxima at 599.31 K and 787.71 K, with corresponding weight loss rates of 0.753%·K^−1^ and 0.068%·K^−1^, respectively. The TG curve also demonstrates three distinct stages of mass loss.

The first stage (320 K to 622.91 K) exhibits a significant cooling reaction with a mass loss of 30.30%, corresponding to the primary thermal decomposition of KAl(OH)_2_CO_3_. This stage involves the substantial release of water and carbon dioxide, playing a crucial role in suffocation and cooling during fire suppression:

$$8\mathrm{KAl}{\left(\mathrm{OH}\right)}_{2}{\mathrm{CO}}_{3}\to 4K_{2}O\cdot {\mathrm{Al}}_{2}O_{3}+6\mathrm{AlO}(\mathrm{OH})+8{\mathrm{CO}}_{2}+5H_{2}O$$

The second stage (622.91 K to 763.71 K) exhibits a mass loss of 6.49%, corresponding to the decomposition of AlO(OH) to generate Al_2_O_3_, while slowly continuing to produce water as follows:

$$\mathrm{AlO}(\mathrm{OH})\to {\mathrm{Al}}_{2}O_{3}+H_{2}O$$

The third stage (763.71 K to 1050 K) shows a mass loss of 5.93%, primarily due to the decomposition of the modified coating film structure on the surface of M-UDWP.

The DSC results, as shown in Figure 6b, indicate that M-UDWP absorbs heat (157.8 J/g) during the first stage of pyrolysis, aiding in the reduction of temperature in the fire zone.

**Figure 7:**
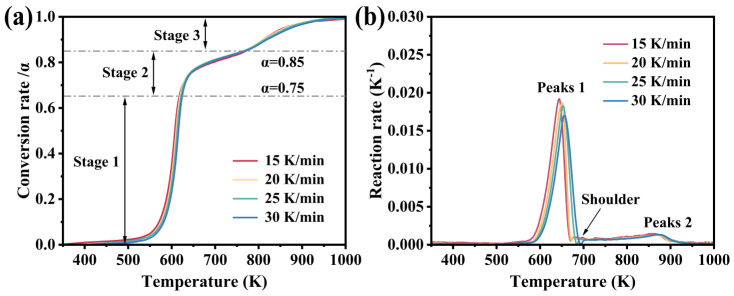
The conversion rate (**a**) and reaction rate (**b**) of M-UDWP change with temperature.

In addition, the curves of the thermal decomposition conversion rate of M-UDWP as a function of temperature under the nitrogen atmosphere at heating rates of 15, 20, 25, and 30 K/min are depicted in Figure 7a. The conversion rate curves exhibit two inflection points at *α* = 0.65 and *α* = 0.85, similar to the TG curves. The reaction rate curves, shown in Figure 7b, resemble the derivative thermogravimetric trends, displaying two peaks and one shoulder, thereby delineating the thermal decomposition process of M-UDWP into three stages. Across the four heating rates, the differences in peak reaction rates, shoulder reaction rates, stage reaction rates, and total reaction rates are minimal. However, the reaction rates at the first peak, shoulder, and second peak decrease with increasing heating rates, as summarized in Table 2.

### 3.3. Thermal Pyrolysis Kinetic

**Figure 8:**
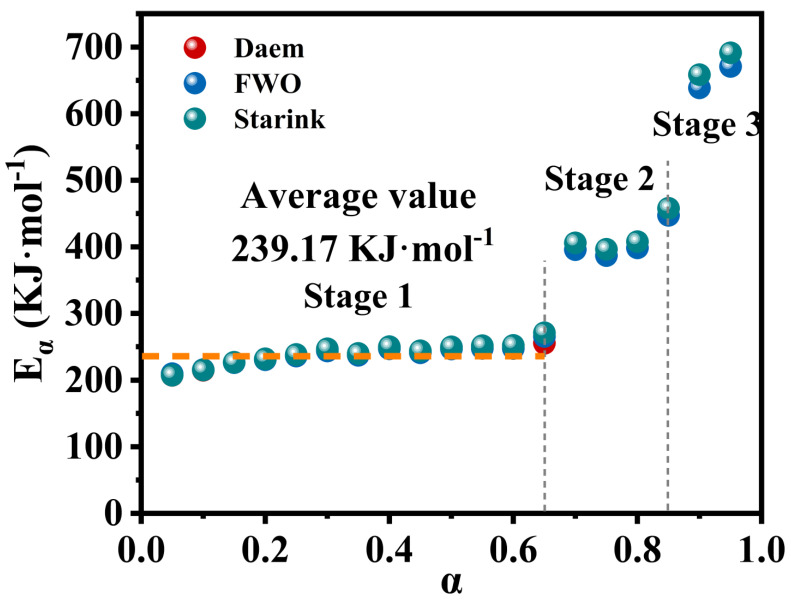
*E_α_* as a function of *α* obtained via Daem, FWO, and Starink methods of M-UDWP.

Krasnyansky [40] identified that activation energy is a key parameter in understanding the fundamental physical and chemical mechanisms by which dry powder extinguishants influence flames. Subtle changes in the activation energy (*E_α_*) during specific decomposition stages may indicate a single-step reaction process. Consequently, analyzing the variations in activation energy during thermal decomposition is of significant importance. Figure 8 illustrates the changes in E values of M-UDWP at different conversion rates (*α*) calculated using three model-free methods. At uniform conversion rates (*α*), the differences in activation energy values derived from the three model-free methods are similarly minimal, allowing the reaction stages to be divided into three phases: Stage 1 (0.05 ≤ *α* ≤ 0.65), Stage 2 (0.65 ≤ *α* ≤ 0.85), and Stage 3 (0.85 ≤ *α* ≤ 0.95), which correspond well with the phases delineated by the conversion rate curves. The activation energy in the first stage incrementally increases and can be treated as a single-step reaction process. Notably, the average activation energy for the first stage is 239.17 kJ/mol, which is lower than the previously calculated activation energy of 357.67 kJ/mol for the first stage of UDWP decomposition [19]. This lower activation energy suggests that the oleophobic/hydrophobic-modified M-UDWP has a reduced energy barrier for thermal decomposition in a fire scenario, enabling rapid pyrolysis and efficient early-stage fire suppression.

Table 3 presents the pre-exponential factor (ln*A*) and activation energy (*E_α_*) for three stages of M-UDWP, calculated using the CR method with closely matching reaction mechanisms. Table S2 lists the ln*A* and *E_α_* values for M-UDWP calculated using the CR method based on these 36 reaction models. Among these, the ln*A* and *E_α_* values calculated based on reaction models No.14 are meaningless. The results indicate that under the conditions of 15, 20, 25, and 30 K/min, the ln*A* and *E_α_* values for M-UDWP are similar. The average *E_α_* value of 223.02 kJ·mol^−1^ obtained based on the No.32 reaction model (*g*(*α*) = [(1 − *α*)^1/3^ − 1]^2^) is the closest to the average *E_α_* value of 239.17 kJ·mol^−1^ obtained by Daem, FWO, and Starink methods. Therefore, the No.32 reaction model, which corresponds to the chemical reaction mechanism, can characterize the thermal degradation of M-UDWP in the first stage. Therefore, a three-dimensional diffusion growth mechanism plausibly describes the first stage of M-UDWP pyrolysis. This mechanism accounts for the spontaneous filling of internal voids within the solid at high temperatures, reducing porosity and increasing the resistance of water vapor and carbon dioxide diffusion through the internal voids to the external surface, thereby affecting the reaction rate.

**Figure 9:**
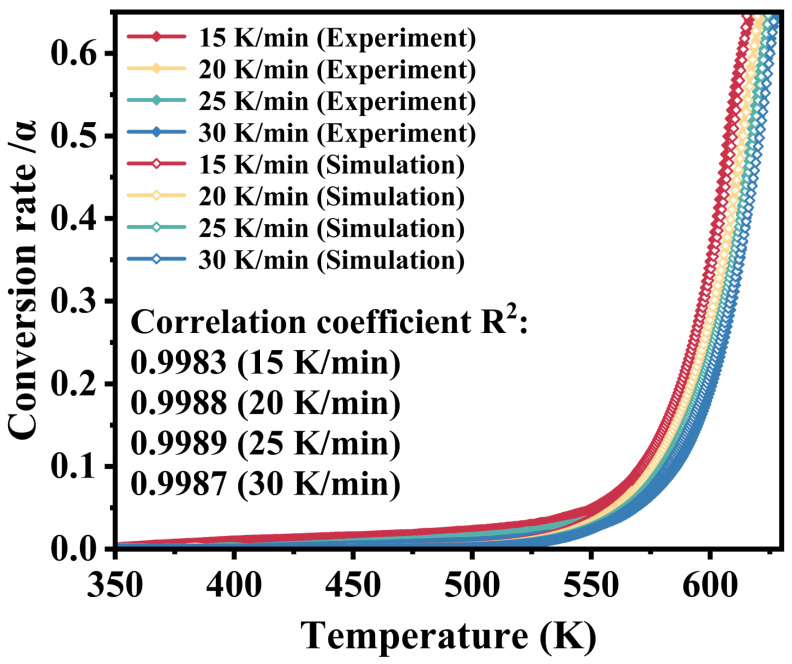
The experimental curve compared with the predicted curve of M-UDWP.

The experimentally measured and predicted *α* values as a function of temperature for M-UDWP are displayed in Figure 9. Within the corresponding range, the predicted data align well with the experimental data, with a correlation coefficient (R^2^) exceeding 0.998, thereby demonstrating that the derived kinetic parameters reliably predict the first-stage thermal decomposition behavior of M-UDWP under a nitrogen atmosphere.

The relationship between *α* and temperature at this stage is described by Equation (10):

(10) $$\alpha =1-[{\left(\frac{m(n-2)e^{-n}}{n^{3}}\right)}^{\frac{1}{2}}+1{]}^{3}$$

where m is defined as Equation (11), and n is defined as Equation (12).

(11) $$m=\frac{AE_{\alpha }}{\beta R}$$

(12) $$n=\frac{E_{\alpha }}{RT}$$

**Figure 10:**
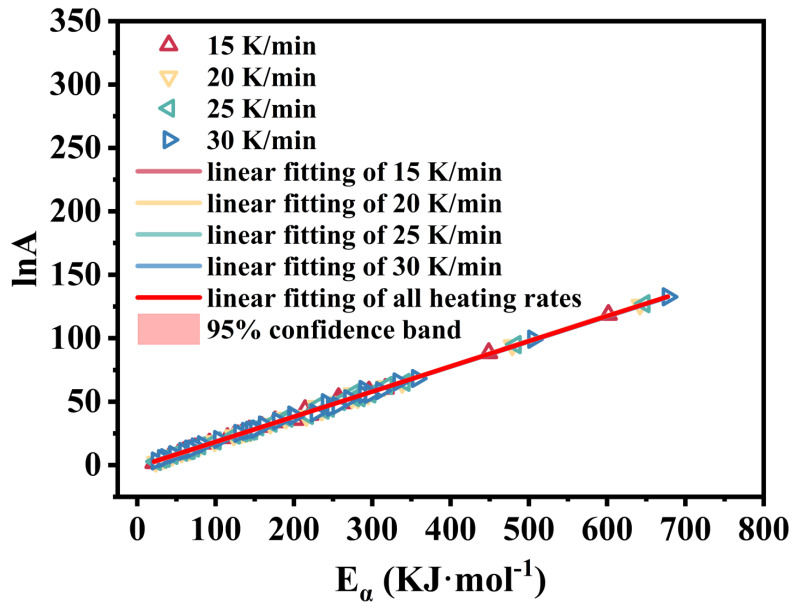
The isokinetic relationships (InA vs. *E_α_*) obtained by the Coats-Redfern method for different heating rates of M-UDWP.

As shown in Figure 10, the compensation effect linear fit between ln*A* and *E_α_* at four different heating rates, as well as the overall linear fit, yield R^2^ values exceeding 0.99. This demonstrates that the 36 model functions selected in this study align well with the thermal decomposition process of M-UDWP. Relevant information on the KCE of M-UDWP is presented in Table 4. The iso conversional temperature (T_iso_) for the first stage is 606.9 K, corresponding to the decomposition temperature range of 350–666 K and indicating the feasibility of the model function used in the CR method.

The linear relationship between ln*A* and *E_α_* is expressed as Equation (13):

(13) $$\ln A=-1.43+0.20E_{\alpha }$$

**Figure 11:**
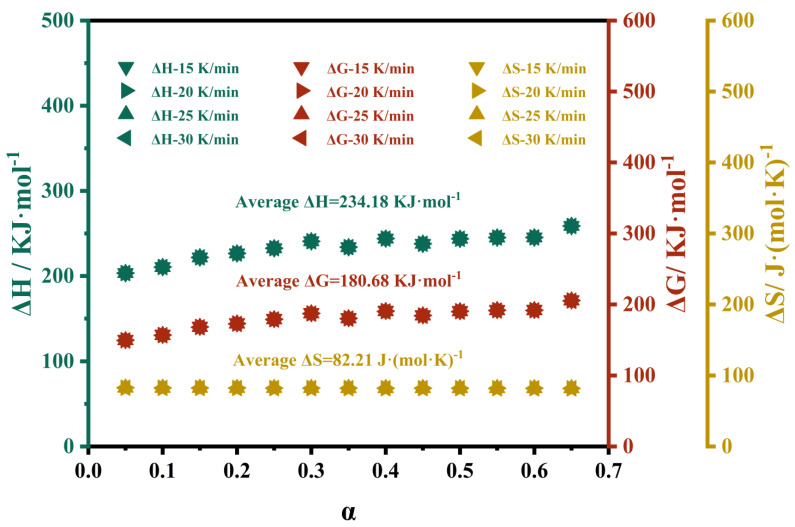
Δ*H*, Δ*G*, and Δ*S* as functions of *α* at 15, 20, 25, and 30 K/min.

Thermodynamic parameters such as Δ*H*, Δ*G*, and Δ*S* are commonly used to analyze the thermal decomposition behavior of solids. Among these parameters, Δ*H* is used to assess the favorability of the thermal transformation process [41], Δ*G* is used to evaluate the feasibility and direction of the reaction [28], and Δ*S* is used to determine the degree of disorder in the thermal transformation process [35]. At heating rates of 15, 20, 25, and 30 K/min, the values of Δ*H*, Δ*G*, and Δ*S* as functions of *α* are shown in Figure 11. The results indicate that for M-UDWP, within the range of 0 ≤ *α* ≤ 0.65, the heating rate has little impact on the average values of Δ*H*, Δ*G*, and Δ*S*, which are 234.18 kJ·mol^−1^, 180.68 kJ·mol^−1^, and 82.21 J·(mol·K)^−1^, respectively, in the first stage of decomposition.

The positive value of Δ*H* indicates that the thermal degradation of M-UDWP in nitrogen is an endothermic process. The constancy of the Δ*H* values suggests that the energy required for the thermal decomposition of M-UDWP remains relatively stable in the first stage of decomposition. In this study, the average Δ*H* of M-UDWP differs from the *E_α_* value by 4.99 kJ·mol^−1^. This indicates that the energy gap between the activated state and the transition state in the reaction is not significant, implying a relatively low activation energy barrier for decomposition. Consequently, when injected into a fire scene, M-UDWP can more readily absorb heat and decompose, thereby inhibiting fire spread.

Δ*G* represents the overall increase in the energy of the reaction system during the thermal transformation. A higher Δ*G* value corresponds to a lower likelihood of the reaction occurring, whereas a lower Δ*G* value indicates a higher likelihood of the reaction taking place. As shown in Figure 11, the constancy of the Δ*G* values suggests that the feasibility of M-UDWP decomposition remains relatively stable in the first stage of decomposition. Moreover, the positive value of Δ*G* indicates that the thermal degradation of M-UDWP in nitrogen may involve non-spontaneous reactions.

As illustrated in Figure 11, the Δ*S* value of M-UDWP remains constant within the range of 0.1 ≤ *α* ≤ 0.65, indicating that the disorder of the reaction system also remains unchanged. The Δ*S* value for M-UDWP can be considered constant for a single-step reaction within the range of 0.1 ≤ *α* ≤ 0.7, which is consistent with the previously discussed results of the variation of E with *α*.

**Figure 12:**
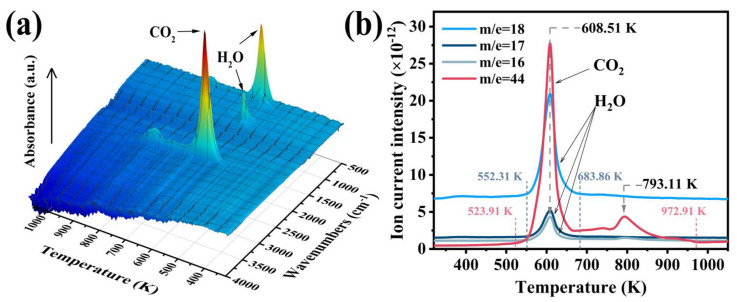
TG-FTIR test results (**a**) and TG-MS test results (**b**) of volatile products produced during the M-UDWP heating process.

To further elucidate the role of volatile products generated during the thermal decomposition of M-UDWP in inhibiting combustion, this study monitored these volatiles using online TG-FTIR-MS at a heating rate of 10 K/min under a nitrogen atmosphere. As illustrated in Figure 12a, the TG-FTIR data reveal the infrared spectra of volatile products produced during the thermal decomposition of M-UDWP. At 552 K, absorption peaks are observed in the wavelength ranges of 1100 cm^−1^ to 1250 cm^−1^, 1400 cm^−1^ to 1900 cm^−1^, and 3500 cm^−1^ to 4000 cm^−1^, which are attributed to the generation of H_2_O [42,43]. The absorption peak at 3580 cm^−1^ arises from the symmetric and asymmetric stretching vibrations of hydroxyl bonds. Additionally, the absorption peak at 1520 cm^−1^ is due to the in-plane bending vibration of oxygen–hydrogen bonds. At 686 K and 880 K, the absorption peak at 2370 cm^−1^, within the temperature range of 523.91 K to 972.91 K, significantly intensifies, reaching peak intensities at 609 K and 793 K. This absorption peak is attributed to the asymmetric stretching vibrations of carbon dioxide [43], indicating the generation of CO_2_ within these temperature ranges.

Further analysis of the TG-MS curves in Figure 12b reveals the presence of H_2_O throughout the entire temperature range, gradually increasing at 552.31 K, peaking at 608.51 K, and then declining, returning to initial levels at 683.86 K. In contrast, CO_2_ production occurs within the temperature range of 523.91 K to 972.91 K, with peaks observed at 608.51 K and 793.11 K, which is consistent with the mass loss curve of M-UDWP discussed in Section 3.3. Unlike the pyrolysis of M-UBDP, the pyrolysis of M-UDWP produces more CO_2_ than H_2_O. It can thus be concluded that the gaseous CO_2_ generated during pyrolysis is the primary inhibitory volatile product in the fire suppression process of M-UDWP [44].

### 3.4. Fire Extinguishing Performance of M-UDWP

**Figure 13:**
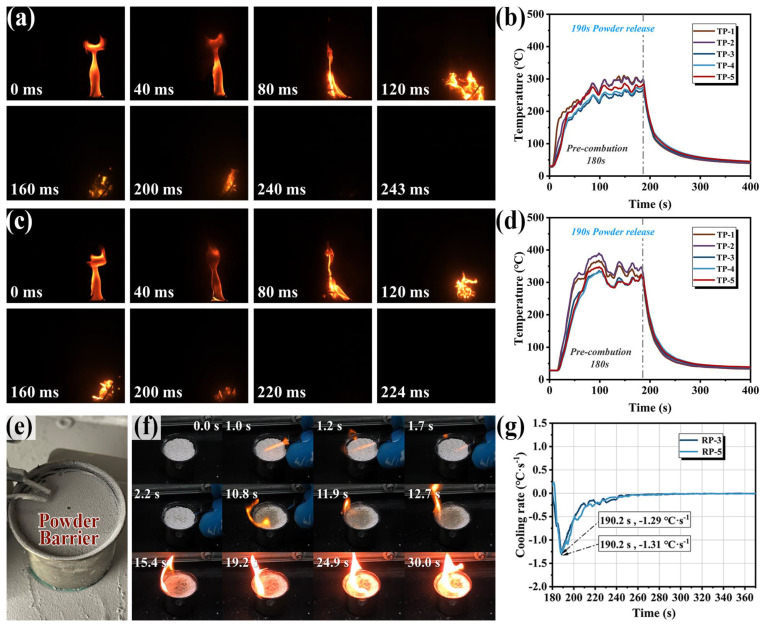
High-speed photography of the fire extinguishing process of #PR-3 flame (**a**) and #PR-5 flame (**c**); fire extinguishing temperature curve of #PR-3 flame (**b**) and #PR-5 flame (**d**); combustible surface after flame extinguishing with M-UDWP (**e**); M-UDWP re-ignition time delay test high-speed photography (**f**); and cooling rate curve of #PR-3 flame and #PR-5 flame (**g**).

Fire suppression experiments were conducted on typical combustibles RP-3 and RP-5 aviation kerosene within aircraft engine nacelles. As illustrated in Figure 13a, the suppression of RP-3 flames by M-UDWP in a confined space was analyzed. At 40 ms, after the extinguishing agent was discharged, air within the space was initially blown towards the flame region, promoting the combustion of the flammable liquid and resulting in flame elongation. At 80 ms, oxygen in the space was consumed by combustion and diluted by nitrogen from the extinguishing agent, causing the flame to flicker. At 120 ms, the flame tilted towards the direction of the extinguishing agent jet due to rapid heat absorption by the agent, cooling and suppressing the flame on the side away from the baffle, forcing the flame to move towards the hotter metal baffle. The high oxygen concentration in the vortex area created by the agent jet at the baffle also facilitated this movement. The high-temperature and the high-oxygen region near the baffle caused the flame to brighten, posing a significant challenge for extinguishing fires behind physical obstructions in aircraft engine compartments. At 160 ms, as the overall temperature decreased and the concentrations of oxygen and flammable vapors dropped, the combustion became dispersed and was gradually suppressed, with complete flame extinction occurring at 240 ms. Figure 13c shows high-speed photography of M-UDWP suppressing RP-5 flames, with complete extinction achieved at 224 ms, following a similar process to that of RP-3.

Figure 13b,d depict the temperature variations during the suppression of RP-3 and RP-5 flames by M-UDWP. After pre-burning for 180 s, the vertical flame temperatures stabilized. From the cooling rate in Figure 13g, the maximum cooling rate of −1.31 °C/s was reached at 190.2 s during the suppression of RP-3 flames, and a similar maximum cooling rate of −1.29 °C/s was observed at 190.2 s during the suppression of RP-5 flames. The cooling rates for extinguishing both types of aviation kerosene flames were essentially identical, with the times to reach the peak cooling rates corresponding to the recorded flame extinction times. The minimum extinguishing concentration (MEC) of M-UDWP for RP-3 flames was recorded at 25.9 g/m^3^, and for RP-5 flames at 23.4 g/m^3^ by the photoelectric sensor.

Figure 13e shows the morphology of the fuel surface after M-UDWP extinguished the aviation kerosene fire. Evidently, due to its oleophobic properties, M-UDWP floated on the surface of the aviation kerosene. In practical operations, the fire suppression system would continuously discharge the agent, allowing more powder to float on the flammable liquid surface and form a fire-retardant layer. This layer can prevent the evaporation of the flammable liquid and the generation of combustible vapors, thus preventing re-ignition. To evaluate the re-ignition prevention capability, an anti-re-ignition test was conducted by spreading 2 g of M-UDWP on the surface of the aviation kerosene and measuring the RTD. The high-speed photography of the M-UDWP re-ignition time delay test is shown in Figure 13f. M-UDWP floated on the surface and was re-ignited at an RTD of 10.8 s. After ignition, the flame spread across the entire liquid surface in 24.9 s. This indicates that even if the flammable liquid re-ignites, M-UDWP floating on the surface of aviation kerosene can effectively prevent the re-ignition and spread of the fire within the aircraft engine nacelle.

In terms of powder flame retardant properties, Olga Gaidukova’s study noted that CO_2_ Hydrate requires a release density of at least 1.3 kg/m^2^ to stop combustion [45], whereas, after calculation, M-UDWP at a release density of 1.59 kg/m^2^ effectively prevents the re-ignition of liquid fuel. While M-UDWP’s flame-suppressing efficiency is slightly lower than CO_2_ Hydrate, M-UDWP has higher thermal stability, making it more suitable for storage in high-temperature environments of aircraft equipment bays, rendering it an ideal fire extinguishing agent for aviation engines.

**Scheme 2:**
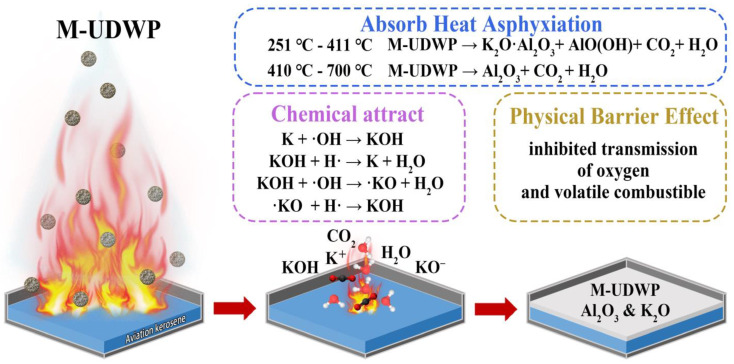
The fire extinguishing and re-ignition prevention mechanisms of M-UDWP.

Scheme 2 discusses the fire extinguishing mechanism of M-UDWP. Its extinguishing effect encompasses both physical and chemical processes. When exposed to heat in a fire scenario, M-UDWP undergoes thermal decomposition, absorbing a substantial amount of heat and generating inert gases and metal ion radicals, which play a crucial role in the extinguishing process. M-UBDP, with its high thermal stability, can absorb more heat from the combustion zone and ensure sufficient contact between the powder and the combustion interface, thereby enhancing the suppression effect and accelerating the cooling process of the combustion area. Additionally, the surface modification endows the powder with excellent oleophobic properties and flow diffusion capabilities, significantly extending the interaction time of the extinguishing medium with the combustion interface, thus enhancing the suppression of the combustion process.

Between 251 °C and 411 °C, M-UDWP decomposes into K_2_O·Al_2_O_3_, AlO(OH), CO_2_, and H_2_O. Compared to M-UBDP, this allows M-UDWP to absorb heat from the surrounding environment at lower temperature stages (earlier stages of a fire), thereby reducing the fire scene temperature. The significant amount of CO_2_ generated during decomposition creates an inert gas atmosphere in the combustion zone, inhibiting combustion. The decomposed products K_2_O and Al_2_O_3_ also act as flame retardants, preventing fire spread. Between 410 °C and 700 °C, the modified coating of M-UDWP continues to decompose, producing carbon dioxide to further inhibit fire spread.

When a large amount of M-UDWP is directed towards the combustion zone in a gas-solid two-phase flow, it forms a uniform aerosol in the combustion area, maintaining a longer suspension time to suppress combustion. The gaseous CO_2_ and H_2_O can dilute the oxygen concentration and flammable vapor concentration in the combustion zone, thereby reducing the intensity of combustion. Additionally, the decomposition of M-UDWP releases K^+^ radicals that chemically adsorb and attack hydroxyl radicals (OH·), with the resulting compounds KOH and KO· radicals engaging in radical scavenging reactions with H· and OH·. The chemical chain-breaking reactions of M-UDWP are more complex, allowing it to capture more H· and OH· radicals at the combustion interface. On this basis, K^+^ radicals exhibit higher reactivity compared to Mg^2+^ radicals, which explains the superior fire extinguishing performance of M-UDWP.

Moreover, the hydrophobic and oleophobic properties of M-UDWP contribute to its fire suppression capabilities. The hydrophobic nature of M-UDWP maintains the dryness and flowability of the powder, preventing particle agglomeration and clustering. This facilitates uniform dispersion of the powder in space, enhancing extinguishing efficiency. The oleophobic M-UDWP forms a dense powder barrier on the surface of flammable liquids. The M-UDWP powder, along with the reaction products K_2_O and Al_2_O_3_, physically isolates the combustible material from the surrounding oxygen, thereby hindering fire propagation. This powder barrier also impedes the continuous evaporation of flammable vapors from the fuel surface. Such isolation not only suppresses ongoing combustion but also prevents re-ignition by separating the elements of the “fire triangle”—fuel, ignition source, and oxidizer. Additionally, the oleophobic properties of M-UDWP help it adhere to the surface of flammable liquids for extended periods in high-temperature and flame environments, prolonging its cooling and chemical extinguishing effects, thereby enhancing overall fire extinguishing performance.

Based on these mechanisms, the M-UDWP fire extinguishing agent can rapidly extinguish aviation kerosene fires with a smaller dosage in a shorter period of time, while also exhibiting excellent re-ignition prevention properties. This contributes to reducing the negative weight burden on aircraft, ensuring flight safety, and protecting aircraft engines from secondary damage caused by re-ignition.

## 4. Conclusions

In this study, a hydrophobic and oleophobic modification coating was formed on the surface of K-dawsonite ultrafine dry powder (UDWP) using perfluorodecyltrimethoxysilane (PFDTMS) and tetraethyl orthosilicate (TEOS), endowing M-UDWP with hydrophobic and oleophobic properties. The pyrolysis kinetics and fire extinguishing performance of M-UDWP were investigated. The key findings of this study are as follows:

(1) The water contact angle (WCA) and oil contact angle (OCA) of the modified M-UDWP are 135.3° and 128.3°, respectively, with hydrophobicity and oleophobicity ratios of 90.34% and 86.59%.
(2) The first-stage thermal decomposition of M-UDWP can be considered a single-step reaction with thresholds at *α* = 0.65 and *α* = 0.85. The activation energy (E) for the first stage is 239.17 KJ·mol^−1^. The adopted chemical reaction model is three-dimensional diffusion (No.32 *g*(*α*) = [(1 − *α*)^1/3^ − 1]^2^).
(3) For common combustibles in two types of aircraft engine nacelles, the extinguishing times for #RP-3 and #RP-5 flames using M-UDWP are 243 ms and 224 ms, respectively, with MEC values of 25.9 g/m^3^ and 23.4 g/m^3^. The maximum cooling rates are similar. It is evident that #RP-3 fires in aircraft engine nacelles are more challenging to suppress.

Therefore, this study prepared an M-UDWP with superhydrophobicity, oleophobicity, and excellent thermal stability. M-UDWP demonstrated outstanding fire extinguishing performance and effectively prevented fuel re-ignition, showing potential as a replacement for traditional Halon 1301 in aircraft engine compartments.

## Figures and Tables

**Table 1:** Mass ratio of M-UDWP before and after stirring in water and aviation kerosene.

	M_0_	M_1_	M_1_/M_0_
water	10	9.034	90.34%
Aviation Kerosene	10	8.659	86.59%

**Table 2:** Details of the reaction rate of M-UDWP at different heating rates.

Heating Rate (K·min^−1^)	Temperature (K) /Reaction Rate (K^−1^)			Temperature (K) /Average Reaction Rate (K^−1^)			Total Average Reaction Rate (K^−1^)
	Peak 1	Shoulder	Peak 2	Stage 1	Stage 2	Stage 3	
15	644.0 /0.0192	678.4 /0.0097	865.4 /0.0014	350–651 /0.0024	651–787 /0.0016	787–1000 /0.0007	0.0019
20	650.5 /0.0187	677.2 /0.0087	866.5 /0.0013	350–657 /0.0022	657–782 /0.0017	782–1000 /0.0007	0.0018
25	652.7 /0.0183	694.0 /0.0073	869.4 /0.0013	350–661 /0.0021	661–776 /0.0018	776–1000 /0.0007	0.0017
30	656.2 /0.0170	704.2 /0.0062	871.7 /0.0013	350–666 /0.0019	666–792 /0.0019	792–1000 /0.0007	0.0017

**Table 3:** ln*A* and *E_α_* for three stages of M-UDWP, calculated using the CR method with the most closely matching reaction mechanisms.

Heating Rate (K/min)	Kinetic Parameter	No. 32 *g*(*α*) = [(1 − *α*)^1/3^ − 1]^2^
15 K/min	*E_α_* (kJ·mol^−1^)	210.6493
	ln*A* (s^−1^)	37.5603
	R^2^	0.9983
20 K/min	*E_α_* (kJ·mol^−1^)	222.7939
	ln*A* (s^−1^)	40.0954
	R^2^	0.9988
25 K/min	*E_α_* (kJ·mol^−1^)	225.9828
	ln*A* (s^−1^)	40.7834
	R^2^	0.9989
30 K/min	*E_α_* (kJ·mol^−1^)	232.6409
	ln*A* (s^−1^)	42.0824
	R^2^	0.9987
Average value	*E_α_* (kJ·mol^−1^)	223.0200
	ln*A* (s^−1^)	40.1300
	R^2^	0.9987

**Table 4:** Values of “KCE” parameters of M-UDWP acquired utilizing the CR method.

Heating Rate (K/min)	a (1/min)	95% CI of a	B (kJ/mol)	95% CI of b	k_iso_	T_iso_ (K)	R^2^
15	−1.8654	(−2.6183, −1.1126)	0.1548	(0.1960, 0.2032)	0.1478	602.7	0.9975
20	−1.5118	(−2.2660, −0.7576)	0.1986	(0.1952, 0.2019)	0.2205	605.7	0.9978
25	−1.2863	(−2.0406, −0.5321)	0.1978	(0.1945, 0.2011)	0.2641	608.1	0.9978
30	−1.0645	(−1.8199, −0.3092)	0.1968	(0.1937, 0.2000)	0.3449	611.0	0.9980

## Data Availability

Data are contained within the article and Supplementary Materials.

## Supplementary Materials

The following supporting information can be downloaded at: https://www.mdpi.com/article/10.3390/molecules29153500/s1, Table S1: Common solid-state pyrolysis reaction models/mechanisms. Table S2: Calculation results of *E_α_* and ln*A* by CR method based upon thermogravimetric data at 15, 20, 25 and 30 K/min by M-UDWP.
