# Supplementary material for: Study on the Pyrolysis and Fire Extinguishing Performance of High-Temperature-Resistant Ultrafine Dry Powder Fire Extinguishing Agents for Aviation Applications
Source: Molecules. 2024 Jul 26;29(15):3500. doi: 10.3390/molecules29153500 (PMC11314109; doi:10.3390/molecules29153500)
Supplement: Supplementary file 1 [file molecules-29-03500-s001.zip › molecules-3118440-supplementary.pdf]

Table S1 Common solid-state pyrolysis reaction models/mechanisms.

No	$g(\alpha)$	$f(\alpha)$	Rate-determining mechanism
1	$1\text{-}(1\text{-}\alpha)^{2/3}$	$3/2(1-\alpha)^{1/3}$	Chemical reaction
2	$1\text{-}(1\text{-}\alpha)^{1/4}$	$4(1-\alpha)^{3/4}$	Chemical reaction
3	$(1-\alpha)^{-1/2}\text{-}1$	$2(1-\alpha)^{3/2}$	Chemical reaction
4	$(1-\alpha)^{-1}\text{-}1$	$(1-\alpha)^{2}$	Chemical reaction
5	$(1\text{-}\alpha)^{-2}\text{-}1$	$1/2(1-\alpha)^{3}$	Chemical reaction
6	$(1\text{-}\alpha)^{-3}\text{-}1$	$1/3(1-\alpha)^{4}$	Chemical reaction
7	$1\text{-}(1\text{-}\alpha)^{2}$	$1/2(1-\alpha)$	Chemical reaction
8	$1\text{-}(1\text{-}\alpha)^{3}$	$1/3(1-\alpha)^{2}$	Chemical reaction
9	$1\text{-}(1\text{-}\alpha)^{4}$	$1/4(1-\alpha)^{3}$	Chemical reaction
10	$\alpha^{3/2}$	$2/3\alpha^{-1/2}$	Nucleation
11	$\alpha^{1/2}$	$2\alpha^{1/2}$	Nucleation
12	$\alpha^{1/3}$	$2\alpha^{1/2}$	Nucleation
13	$\alpha^{1/4}$	$4\alpha^{3/4}$	Nucleation
14	$ln\alpha$	A	Nucleation
15	$\text{-}ln(1\text{-}\alpha)$	$1-\alpha$	Assumption of random nucleation and subsequent growth
16	$[\text{-}ln(1\text{-}\alpha)]^{2/3}$	$3/2(1-\alpha)[\text{-}ln(1\text{-}\alpha)]^{1/3}$	Assumption of random nucleation and subsequent growth
17	$[\text{-}ln(1\text{-}\alpha)]^{1/2}$	$2(1-\alpha)[\text{-}ln(1\text{-}\alpha)]^{1/2}$	Assumption of random nucleation and subsequent growth
18	$[\text{-}ln(1\text{-}\alpha)]^{1/3}$	$3(1-\alpha)[\text{-}ln(1\text{-}\alpha)]^{2/3}$	Assumption of random nucleation and subsequent growth
19	$[\text{-}ln(1\text{-}\alpha)]^{1/4}$	$4(1-\alpha)[\text{-}ln(1\text{-}\alpha)]^{3/4}$	Assumption of random nucleation and subsequent growth
20	$[\text{-}ln(1\text{-}\alpha)]^{2}$	$1/2(1-\alpha)[-ln(1-\alpha)]^{-1}$	Assumption of random nucleation and subsequent growth
21	$[\text{-}ln(1\text{-}\alpha)]^{3}$	$1/3(1-\alpha)[\text{-}ln(1\text{-}\alpha)]^{2}$	Assumption of random nucleation and subsequent growth
22	$[\text{-}ln(1\text{-}\alpha)]^{4}$	$1/4(1-\alpha)[-ln(1-\alpha)]^{-3}$	Assumption of random nucleation and subsequent growth
23	$ln\alpha/(1\text{-}\alpha)$	$\alpha/(1-\alpha)$	Nucleation branching reaction
24	α	1	Contracting disk

25	$1-(1-\alpha)^{1/2}$	$2(1-\alpha)^{1/2}$	Contracting sphere (spherical symmetry)
26	$1-(1-\alpha)^{1/3}$	$3(1-\alpha)^{2/3}$	Contracting sphere (spherical symmetry)
27	α^2	$1/2\alpha$	One-dimensional diffusion
28	$[1-(1-\alpha)^{1/2}]^{1/2}$	$4\{(1-\alpha)[1-(1-\alpha)]^{1/2}\}^{1/2}$	Two-dimensional diffusion
29	$\alpha+(1-\alpha)ln(1-\alpha)$	$[-ln(1-\alpha)]^{-1}$	Two-dimensional diffusion
30	$[1-(1-\alpha)^{1/3}]^2$	$(3/2)(1-\alpha)^{2/3}[1-(1-\alpha)^{1/3}]-1$	Three-dimensional diffusion, spherical symmetry
31	$1-2/3\alpha-(1-\alpha)^{2//3}$	$(3/2)[(1-\alpha)^{-1/3}-1]-1$	Three-dimensional diffusion, cylindrical symmetry
32	$((1-\alpha)^{1/3}-1)^2$	$(3/2)[(1-\alpha)^{-1/3}-1]^{-1}(3/2)(1-\alpha)^{4/3}[(1-\alpha)^{-1/3}-1]^{-1}$	Three-dimensional diffusion
33	$((1+\alpha)^{1/3}-1)^2$	$(3/2)(1+\alpha)^{2/3}[(1+\alpha)^{1/3}-1]^{-1}$	Three-dimensional diffusion
34	$1+2/3\alpha-(1+\alpha)^{2/3}$	$(3/2)[(1+\alpha)^{-1/3}-1]^{-1}$	Three-dimensional diffusion
35	$((1+\alpha)^{-1/3}-1)$^2	$(3/2)(1+\alpha)^{4/3}[(1+\alpha)^{-1/3}-1]^{-1}$	Three-dimensional diffusion
36	$(1-(1-\alpha)^{1/31/2}$	$6(1-\alpha)^{2/3}[1-(1-\alpha)^{1/3}]^{1/2}$	Three-dimensional diffusion

Table S2 Calculation results of E_α and lnA by CR method based upon thermogravimetric data at 15, 20 ,25and 30 K/min by M-UDWP.

No	g(α)	15K/min		20K/min		25K/min		30K/min	
		E_α (kJ·mol^{-1})	lnA (s^{-1})	E_α (kJ·mol^{-1})	lnA (s^{-1})	E_α (kJ·mol^{-1})	lnA (s^{-1})	E_α (kJ·mol^{-1})	lnA (s^{-1})
1	$1-(1-\alpha)^{2/3}$	132.29	24.73	140.12	26.49	142.17	27.02	146.45	27.93
2	$1-(1-\alpha)^{1/4}$	145.73	26.65	154.31	28.54	156.56	29.09	161.26	30.07
3	$(1-\alpha)^{-1/2}-1$	172.47	33.06	182.54	35.22	185.19	35.83	190.71	36.95
4	$(1-\alpha)^{-1}-1$	192.11	37.94	203.27	40.29	206.21	40.95	212.35	42.16
5	$(1-\alpha)^{-2}-1$	250.56	47.83	264.13	50.63	267.71	51.37	275.19	52.81
6	$(1-\alpha)^{-3}-1$	284.07	58.47	300.34	61.75	304.64	62.6	313.62	64.28
7	$1-(1-\alpha)^{2}$	95.84	17.91	101.63	19.30	103.14	19.75	106.30	20.48
8	$1-(1-\alpha)^{3}$	74.49	13.59	79.09	14.77	80.28	15.17	82.78	15.79
9	$1-(1-\alpha)^{3}$	57.48	10.04	61.13	11.05	62.06	11.41	64.05	11.95
10	$\alpha^{3/2}$	188.35	36.13	199.27	38.45	202.14	39.09	208.13	40.28
11	$\alpha^{1/2}$	56.16	9.47	59.77	10.47	60.70	10.84	62.66	11.37
12	$\alpha^{1/3}$	34.13	4.73	36.52	5.51	37.13	5.84	38.42	6.26
13	$\alpha^{1/4}$	23.12	2.22	24.89	2.90	25.34	3.20	26.30	3.57
14	$\ln(a)$	-	-	-	-	-	-	-	-
15	$-\ln(1-a)$	99.55	18.72	105.56	20.15	107.13	20.61	110.43	21.36
16	$(-\ln(1-a))^{2/3}$	72.18	13.05	76.68	14.21	77.84	14.61	80.30	15.23
17	$(-\ln(1-a))^{1/2}$	44.81	7.22	47.79	8.11	48.56	8.46	50.18	8.93
18	$(-\ln(1-a))^{1/3}$	31.13	4.18	33.35	4.94	33.91	5.25	35.12	5.67
19	$(-\ln(1-a))^{1/4}$	318.49	62.70	336.65	66.32	341.44	67.24	351.42	69.09
20	$(-\ln(1-a))^{2}$	482.70	95.23	509.96	100.50	517.17	101.76	532.17	104.42
21	$(-\ln(1-a))^{3}$	646.90	127.64	683.27	134.55	692.90	136.15	712.92	139.64
22	$(-\ln(1-a))^{4}$	122.26	22.97	129.52	24.63	131.42	25.13	135.40	26.00

23	$\frac{ln\alpha}{1-\alpha}$	137.55	25.58	145.66	27.39	147.79	27.93	152.24	28.87
24	α	142.96	26.34	151.38	28.20	153.59	28.75	158.21	29.72
25	$1-(1-\alpha)^{1/2}$	254.44	49.16	269.02	52.13	272.87	52.91	280.87	54.43
26	$1-(1-\alpha)^{1/3}$	63.81	10.84	67.84	11.92	68.89	12.30	71.08	12.87
27	α^2	273.93	52.60	289.61	55.77	293.74	56.59	302.35	58.21
28	$(1-(1-\alpha)^{1/2})^{1/2}$	295.85	55.73	312.75	59.12	317.20	59.99	326.49	61.72
29	$\alpha+(1-\alpha)ln(1-\alpha)$	281.22	52.63	297.30	55.88	301.54	56.72	310.37	58.37
30	$(1-(1-\alpha)^{1/3})^2$	342.42	65.55	361.91	69.41	367.06	70.38	377.79	72.34
31	$1-2/3\alpha-(1-\alpha)^{2/3}$	241.84	42.12	255.17	44.86	258.67	45.60	265.98	47.00
32	$((1-\alpha)^{1/3}-1)^2$	210.65	37.56	222.79	40.10	225.98	40.78	232.64	42.08
33	$((1+\alpha)^{1/3}-1)^2$	244.13	43.68	257.87	46.50	261.48	47.25	269.01	48.69
34	$1+2/3\alpha-(1+\alpha)^{2/3}$	66.52	11.24	70.70	12.35	71.79	12.74	74.07	13.32
35	$((1+\alpha)^{-1/3}-1)^2$	201.95	35.75	215.91	38.67	217.89	39.12	227.92	41.10
36	$(1-(1-\alpha)^{1/3})^{1/2}$	61.63	10.19	66.29	11.40	66.920	11.70	70.35	12.54
